# Spinal cord stimulation alleviates intractable pain due to malignant pleural mesothelioma: a case report

**DOI:** 10.1186/s40981-020-00386-9

**Published:** 2020-10-06

**Authors:** Aiko Maeda, Masatsugu Watanabe, Chiaki Saigano, Shoko Nakayama, Ken Yamaura

**Affiliations:** 1 Operating rooms, Kyushu University Hospital, 3-1-1 Maedashi Higashi-ku, Fukuoka City, Fukuoka, 812-8582 Japan; 2 Department of Anesthesiology and Critical Care Medicine, Kyushu University Hospital, Fukuoka, Japan; 3 Department of Anesthesiology and Critical Care Medicine, Kyushu University Graduate School of Medicine, Fukuoka, Japan

**Keywords:** Spinal cord stimulation, Malignant pleural mesothelioma, Neuropathic pain, Cancer pain

## Abstract

**Background:**

Patients with malignant pleural mesothelioma (MPM) frequently complain of intractable pain that is resistant to conservative treatments. Although spinal cord stimulation (SCS) may be promising in the alleviation of such devastating pain, the effects of SCS on MPM-associated pain and the appropriate timing of its application remain unknown.

**Case presentation:**

A 66-year-old man diagnosed with MPM presented with severe neuropathic pain due to rapid progression of the tumor to the intercostal nerves. The patient immediately decided to receive SCS implantation and burst stimulus, which relieved the conservative therapy-resistant pain and improved his sleep and daily activities.

**Conclusion:**

This report suggests that the execution of SCS as soon as possible may help to alleviate MPM symptoms. Since MPM extends aggressively to the thorax and nerves that cause mixed nociceptive and/or neuropathic pain, appropriate pain management requires the proper assessment of the etiology by an expert in pain management.

## Background

Caused by exposure to asbestos, malignant pleural mesothelioma (MPM) is a cancer that affects the pleural lining. It has a considerable symptom burden, progresses rapidly, and a poor prognosis [1, 2]. MPM frequently inflicts both nociceptive and neuropathic pain due to direct infiltration into the chest wall and nerves. Despite the use of multiple analgesics, its aggressiveness often poses major challenges in pain management [3, 4]. Therefore, rapid and adequate pain assessment in patients with MPM by an expert in pain management is essential for guiding the appropriate choice of treatment, as neuropathic pain may be resistant to opioids. Spinal cord stimulation (SCS) is a procedure performed to relieve refractory chronic neuropathic pain, such as opioid resistant pain [5]. Here, we report a successful SCS procedure for a patient with MPM with refractory neuropathic pain secondary to tumor infiltration into the intercostal nerves.

This article adheres to the applicable Enhancing the Quality and Transparency Of health Research (EQUATOR) guideline. Written informed consent was obtained from the patient for publication of the case report.

## Case presentation

A 66-year-old man with a history of occupational asbestos exposure was referred to the Department of Thoracic Oncology 4 years ago. On contrast-enhanced computed tomography (CT) of the chest, a mass was observed in the left pleura. The pathological diagnosis was sarcomatoid MPM. The detailed examination revealed the cancer to be a T4 N0 M0 stage IV MPM; cisplatin and pemetrexed as the first-line therapy and an immune checkpoint inhibitor as the second-line therapy were administered. Over the past years, the patient had received appropriate conservative treatment for pain, including opioids and other analgesics. Although the left thoracic pain recurred 6 months prior to admission, it disappeared after palliative radiation therapy. For a while, since then, his pain was well-controlled with the use of strong opioids. About a month before visiting us, the pain recurred and failed to respond to the administration of pregabalin, amitriptyline, and controlled-release (CR) and immediate-release (IR) oxycodone (approximately 320 mg total oral morphine equivalents). He was referred to our pain management clinic to be evaluated for SCS.

The patient presented to us with severe pain at the banded region from the left lower scapula to the left axilla and lower edge of the breast. On the numerical rating scale (NRS) ranging from 0 to 10, his maximum pain was rated at 8. The spontaneously recurring, intense paroxysmal sharp-electrical and shooting pain occurred in an area of sensory impairment and lasted for at least 2 h. Administration of IR oxycodone over 10 times a day did not improve his condition. The pain severely interrupted the patient’s sleep and daily activities. In contrast, the pain in the wide left thoracic area responded well to opioid analgesics. Therefore, it was considered that his pain consisted of mixed pre-existing nociceptive and new-onset neuropathic pain. Given that the patient was deemed a good candidate for SCS, we decided to perform a SCS trial and implantation. At the time of implantation, his quantitative prognosis was expected to be about six months to a year.

**Fig. 1:**
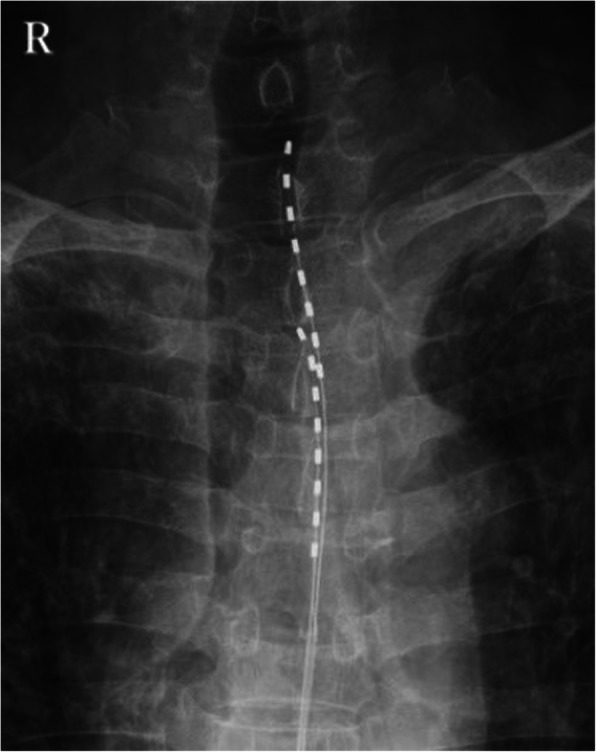
Fluoroscopy image of the spinal cord stimulator. Two electrodes were introduced into the epidural space from the T12/L1 intervertebral space. The tip of the first electrode was positioned slightly to the left of the midline at the level of T1. The second electrode was on the caudal side of the first electrode

**Fig. 2:**
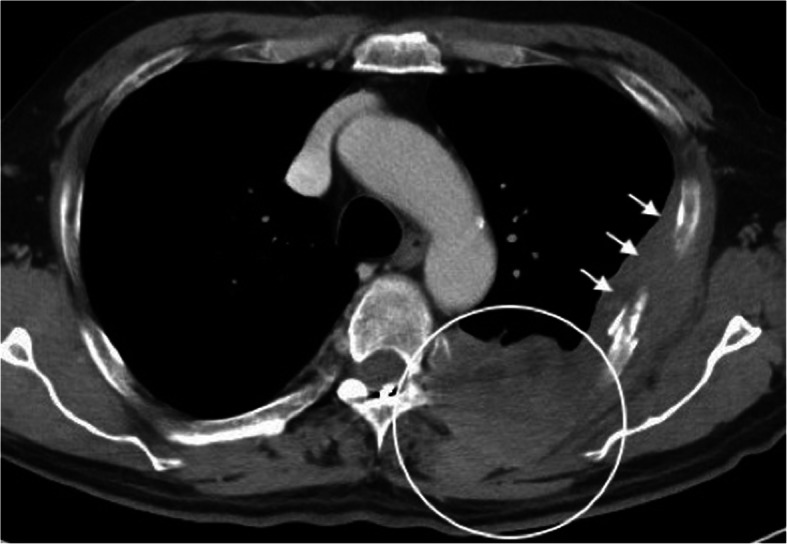
Axial slice of a CT scan showing a heterogeneous mass infiltrating the posterior thoracic tissue (in the white circle), and pleural thickening (white arrows)

Two Tuohy needles were inserted into the epidural space of the vertebral level T12/L1. Each electrode lead (Octrode, Abbott, Plano, TX, USA) was subsequently introduced into the epidural space. The tip of the first electrode was positioned slightly left of the midline of the T1 vertebrae by fluoroscopic guidance. Similarly, the second electrode was placed on the caudal side of the first lead (Fig. 1). Complete paresthesia coverage, extending from the left scapula to the upper precordial region, was achieved with the stimulation. The patient had a 7-day trial of SCS, and burst SCS proved to be better for him than tonic SCS. Burst SCS involves a stimulation pattern often utilized clinically, consisting of five pulses (1 ms duration) of 500 Hz, with the bursts repeated at 40 Hz. After completing the stimulation trial, and as the patient’s pain intensity improved from 8 to 4 on the NRS, he underwent permanent implantation of an implantable pulse generator (IPG). The IPG (Proclaim Elite 5, Abbott, Plano, TX, USA) was embedded in a subcutaneous pocket in the left gluteal region. The patient reported a significant increase in his daily activities as well as a decrease in sleep disturbance. He could also reduce the intake of total oxycodone (approximately 180 mg total oral morphine equivalents). Repeated chest CTs showed a further increase in the left pleural mass (Fig. 2). About a month after the implantation, the shooting pain had spread, for which we changed some stimulation electrode sites, resulting in prolonged pain relief for the patient’s remaining eight months of his life.

## Discussion

Appropriate management for MPM-associated pain needs prompt and adequate assessment by pain management experts, as well as an understanding of mixed nociceptive-neuropathic pain, as the tumor rapidly and aggressively infiltrates into the chest wall, ribs, nerve roots, intercostal nerves, neurovascular bundle, or a combination of these [3]. Neuropathic pain has some characteristic clinical manifestations, such as the existence of partial sensory loss and burning or shooting pain [6], which have been described as negative predictive prognostic factors in cancer pain therapy [7]. More advanced stages in cancer correlate with increased difficulty in pain control, as around 20% of patients with cancer pain are refractory to opioid therapy. Adjuvant analgesics can be added at any step in the analgesic ladder and are useful in the management of neuropathic pain [8, 9]. Nonetheless, a systematic review by Bennett [10] suggested that the addition of adjuvants does not reduce pain intensity > 1 point on a 0–10 NRS. When cancer pain is resistant to conventional pharmacological therapies, interventional techniques may be employed; according to previous reports, instead of a last resort, these could be utilized at any stage [5]. As for MPM, previous reports have advocated that percutaneous cervical cordotomy (PCC) and peripheral nerve blocks ameliorate challenging pain syndromes [4]. Other interventional techniques for cancer pain include SCS and an implantable drug delivery system that involves placing a subarachnoid catheter for infusing a combination of opioids and adjunct medications directly into the central nervous system [5]. However, PCC is more invasive and is not recommended for patients with poor respiratory or general conditions. In addition, nerve blocks are contraindicated in patients with tumors infiltrating into the intervertebral foramina and intercostal nerves, as is often the case in patients with MPM. We decided to prioritize SCS over other interventional therapies for this patient as it was safer and did not require replenishment of drugs.

Although the mechanism of SCS is not completely understood, several mechanisms have been proposed, such as segmental and supraspinal level mechanisms. These include the reduction of pain stimulation advocated by Wall and Melzack’s gate control theory, the increase of inhibitory neurotransmitter release, and descending modulation [11]. The concept of burst SCS was introduced in 2010 by De Ridder et al. [12]. Burst SCS affects the somatic nervous system without the need for paresthesia overlap in the target area [12, 13]. Basic research using rat models exploring the mechanism of burst SCS-mediated analgesia implicates a significant suppression of pinch-evoked activity of wide dynamic range neurons and controlled mechanical hypersensitivity [14, 15]. A wide body of evidence suggests that burst SCS demonstrates great pain relief in clinical settings, and it appears to interfere with multiple dimensions of pain, including emotional elements [13]. We ultimately chose burst SCS for our patient because of its efficacy and the lesser discomfort caused by it compared to tonic SCS.

Although clinical evidence for SCS in the treatment of cancer pain is still lacking, several case series and case reports in the literature led us to speculate that the SCS’s effect on chest wall cancer pain is effective [16, 17]. Currently, there is evidence that SCS is effective for non-malignant neuropathic pain represented by post-spine surgery syndrome [11]. Cancer-related neuropathic pain caused by tumor infiltration to the nerves has some features in common with the pathogenesis of non-malignant neuropathic pain. There are also other causes of neuropathic pain such as the reaction of cancer cells and immune cells [18]. A previous study indicated that approximately 30% of patients with cancer have neuropathic pain for these various reasons [18]; thus, it is possible that cancer-related pain may be a good candidate for SCS.

Nonetheless, to the best of our knowledge, the effect or appropriate insertion timing of SCS on MPM-associated pain is completely unknown because MPM has different, rapid, and aggressive characteristics compared to other cancers. Our experience with this case recommends that early SCS should be selected for patients with MPM and that the lead be situated to obtain a wide range of stimulation. Further studies are recommended to evaluate the efficacy of SCS in patients with MPM-associate refractory neuropathic pain.

In conclusion, SCS relieved conservative therapy-resistant neuropathic pain in our patient with MPM, thereby improving his sleep and daily activities. When the pain management specialist determines that a patient with MPM has developed intractable neuropathic pain, the implantation of SCS as expeditiously as possible may improve the patient’s symptoms.

## Data Availability

Not applicable

## Abbreviations

CR: Controlled-release
CT: Computed tomography
EQUATOR: Enhancing the quality and Transparency Of health Research
IPG: Implantable pulse generator
IR: Immediate-release
MPM: Malignant pleural mesothelioma
NRS: Numerical rating scale
PCC: Percutaneous cervical cordotomy
SCS: Spinal cord stimulation
